# Myopathy Associated With Dermatan Sulfate-Deficient Decorin and Myostatin in Musculocontractural Ehlers-Danlos Syndrome: A Mouse Model Investigation

**DOI:** 10.3389/fcell.2021.695021

**Published:** 2021-10-11

**Authors:** Yuko Nitahara-Kasahara, Guillermo Posadas-Herrera, Shuji Mizumoto, Aki Nakamura-Takahashi, Yukiko U. Inoue, Takayoshi Inoue, Yoshihiro Nomura, Shin’ichi Takeda, Shuhei Yamada, Tomoki Kosho, Takashi Okada

**Affiliations:** ^1^Department of Biochemistry and Molecular Biology, Nippon Medical School, Tokyo, Japan; ^2^Division of Molecular and Medical Genetics, Center for Gene and Cell Therapy, The Institute of Medical Science, The University of Tokyo, Tokyo, Japan; ^3^Department of Pathobiochemistry, Faculty of Pharmacy, Meijo University, Nagoya, Japan; ^4^Department of Pharmacology, Tokyo Dental College, Tokyo, Japan; ^5^Department of Biochemistry and Cellular Biology, National Center of Neurology and Psychiatry, National Institute of Neuroscience, Kodaira, Japan; ^6^Faculty of Agriculture, Tokyo University of Agriculture and Technology, Fuchu, Japan; ^7^National Institute of Neuroscience, National Center of Neurology and Psychiatry, Kodaira, Japan; ^8^Department of Medical Genetics, Shinshu University School of Medicine, Matsumoto, Japan; ^9^Center for Medical Genetics, Shinshu University Hospital, Matsumoto, Japan; ^10^Division of Clinical Sequencing, Shinshu University School of Medicine, Matsumoto, Japan; ^11^Research Center for Supports to Advanced Science, Shinshu University, Matsumoto, Japan

**Keywords:** Ehlers-Danlos syndrome, dermatan sulfate, dermatan 4-*O*-sulfotransferase 1, decorin, chst14 mutant mouse, myostatin, myopathy

## Abstract

*Carbohydrate sulfotransferase 14* (*CHST14*) encodes dermatan 4-*O*-sulfotransferase 1, a critical enzyme for dermatan sulfate (DS) biosynthesis. Musculocontractural Ehlers-Danlos syndrome (mcEDS) is associated with biallelic pathogenic variants of *CHST14* and is characterized by malformations and manifestations related to progressive connective tissue fragility. We identified myopathy phenotypes in *Chst14*-deficient mice using an mcEDS model. Decorin is a proteoglycan harboring a single glycosaminoglycan chain containing mainly DS, which are replaced with chondroitin sulfate (CS) in mcEDS patients with CHST14 deficiency. We studied the function of decorin in the skeletal muscle of *Chst14*-deficient mice because decorin is important for collagen-fibril assembly and has a myokine role in promoting muscle growth. Although decorin was present in the muscle perimysium of wild-type (*Chst14^+/+^*) mice, decorin was distributed in the muscle perimysium as well as in the endomysium of *Chst14^–/–^* mice. *Chst14^–/–^* mice had small muscle fibers within the spread interstitium; however, histopathological findings indicated milder myopathy in *Chst14^–/–^* mice. Myostatin, a negative regulator of protein synthesis in the muscle, was upregulated in *Chst14^–/–^* mice. In the muscle of *Chst14^–/–^* mice, decorin was downregulated compared to that in *Chst14^+/+^* mice. *Chst14^–/–^* mice showed altered cytokine/chemokine balance and increased fibrosis, suggesting low myogenic activity in DS-deficient muscle. Therefore, DS deficiency in mcEDS causes pathological localization and functional abnormalities of decorin, which causes disturbances in skeletal muscle myogenesis.

## Introduction

The musculocontractural Ehlers-Danlos syndrome (mcEDS) subtype is caused by defective biosynthesis of dermatan sulfate (DS). Most patients with mcEDS have biallelic pathogenic variants in the gene for *carbohydrate sulfotransferase 14* (*CHST14*), which encodes dermatan 4-*O*-sulfotransferase 1 (D4ST1) (mcEDS-*CHST14*), whereas the remaining patients have biallelic pathogenic variants in the DS epimerase gene (Dündar et al., 2009; Miyake et al., 2010; Müller et al., 2013; Brady et al., 2017; Kosho et al., 2020). mcEDS is clinically characterized by craniofacial features, multiple congenital contractures, ocular and visceral malformations, and progressive connective tissue fragility-related manifestations, such as skin hyperextensibility and fragility, joint hypermobility with luxation, progressive spinal and foot deformities, large subcutaneous hematomas, and visceral ruptures (Kosho et al., 2011, 2020; Kosho, 2016). The myopathic process has been suggested in mcEDS because of a reduced amplitude of muscle action potential with normal distal latency time and nerve conduction velocity (Dundar et al., 1997); the muscle phenotypes of a patient with mcEDS-*CHST14* have also been reported (Voermans et al., 2012).

Proteoglycans are the most abundant components of the non-fibrillar extracellular matrix (ECM). They are composed of a protein core to which long, linear, highly sulfated glycosaminoglycan (GAG) chains are covalently attached. Proteoglycans display different functions that are principally mediated by GAG chains (Sugahara et al., 2003). The main sulfated GAG families in muscles are chondroitin sulfate (CS)/DS, heparan sulfate, and keratan sulfate (Handel et al., 2005; Fadic et al., 2006; Hannesson et al., 2007; Zhang, 2010; Negroni et al., 2014). Decorin is a proteoglycan that contains a single GAG chain and plays an important role in the assembly of collagen fibrils, possibly via electrostatic interaction between decorin-DS chains and adjacent collagen fibrils (Iozzo, 1998; Nomura, 2006). The GAG side chain of decorin from the skin fibroblasts of mcEDS-*CHST14* patients contained CS instead of DS (Miyake et al., 2010). Collagen fibrils normally aggregate in line and form collagen fibers, which are round and uniform. Although there were no significant differences in the diameter of collagen fibrils as well as the circularity as an index of shape between *Chst14*^+/+^ and *Chst14*^–/–^ mice, collagen fibrils were scattered and oriented in various directions (Hirose et al., 2021). Furthermore, irregular shapes and sizes of collagen fibrils were detected in decorin-null mice (Danielson et al., 1997), being partially different from those in *Chst14*^–/–^ mice.

Decorin is also characterized as a myokine, which is elevated following exercise in normal muscle, and promotes muscle fiber hypertrophy by competitively binding to inhibit myostatin, a negative regulator of muscle protein synthesis (Ostrowski et al., 1998; Lightfoot and Cooper, 2016). In transgenic models, decorin has been shown to induce upregulation of factors associated with myogenesis, such as MyoD and follistatin (Lightfoot and Cooper, 2016). In addition, transforming growth factor type β (TGF-β), a potent inhibitor of myogenesis like myostatin, is also regulated by complexing with decorin (Florini et al., 1991). Decorin requirement seems to be necessary for myogenesis as decorin expression accelerates skeletal muscle differentiation (Cabello-Verrugio and Brandan, 2007; Brandan and Gutierrez, 2013).

We developed CRISPR/Cas9-genome engineered *Chst14* mutant (*Chst14^–/–^*) mice, which showed a pathological phenotype and shared the typical mcEDS phenotype features, including loss of DS, growth delay, skin fragility, myopathy, reduced muscle function, and thoracic kyphosis. In the present study, we investigated the effects of decorin on spatial distribution and expression in myopathy using *Chst14^–/–^* mice as an mcEDS model.

## Materials and Methods

All experimental procedures were approved by the Experimental Animal Care and Use Committee at the National Center of Neurology and Psychiatry (NCNP) and Nippon Medical School. *Chst*14^−/−^ mice with a 6 base pair (bp) insertion/10 bp deletion (31_40delinsCCACTG) and 1 bp deletion (–1 bp mutant; c.57delG) were developed by CRISPR/Cas9-genome engineering at NCNP (Nitahara-Kasahara et al., 2021b) and were maintained according to the standard protocol for animal care at the NCNP and Nippon Medical School. *Chst*14^−^^/–^ mice were inbred as C57BL/6 and 129svj mixed backgrounds. 129S1/SvImJ and B6C3F1 mice were purchased from Nihon CLEA (Tokyo, Japan) and Japan SLC, Inc. (Shizuoka, Japan), respectively. Age-matched littermate mice were used in all the experiments. Each mouse group contained sex-matched mice (females, *n* = 2; males, *n* = 2).

### Histopathology and Immunohistochemistry

The tibialis anterior (TA) muscle obtained from age- and sex-matched mice was immediately frozen in liquid nitrogen-cooled isopentane. Transverse cryosections (10 μm thickness) were prepared from frozen muscle tissues, stained with hematoxylin and eosin (H&E) using standard procedures, and immunostained for decorin. Muscle cryosections fixed with 1% paraformaldehyde were treated with anti-decorin antibody (monoclonal mouse IgG1 clone 115402, R&D Systems, Minneapolis, MN) or anti-laminin β-1 antibody (Abcam, Cambridge, United Kingdom) as the primary antibody, followed by Alexa 488-conjugated anti-rat IgG antibody (Thermo Fisher Scientific, Waltham, MA) or Alexa 594-conjugated anti-mouse IgG antibody (Thermo Fisher Scientific) as the secondary antibody. The sections were mounted in Vectashield with 4, 6-diamidino-2-phenylindole (Vector Laboratories). Immunofluorescence and H&E staining were visualized using an IX81 fluorescence microscope (Olympus, Tokyo, Japan). For quantification analysis of myofiber size distribution, the myofiber area in H&E images (95-100 fibers) was measured using CellSence software (Olympus). For collagen staining, sirius red staining of cryosections from the TA muscle was performed using a general protocol (Morphotechnology, Sapporo, Japan). Quantitative analysis of the sirius red staining area was performed using CellSence software (Olympus).

### Enzyme-Linked Immunosorbent Assay

Protein expression levels were measured in TA muscle lysate obtained from each mouse using a Quantikine enzyme-linked immunosorbent assay (ELISA), such as mouse myostatin Immunoassay (Thermo Fisher Scientific), mouse collagen type I, and type III Immunoassay (Cloud-clone Corp., Katy, TX), and mouse transforming growth factor-β1 (TGF-β1) Immunoassay (R&D Systems), according to the manufacturer’s recommendations. The final values were normalized to protein concentrations and measured using a Pierce^®^ BCA Protein Assay Kit (Thermo Fisher Scientific).

### Reverse Transcription Polymerase Chain Reaction

Total RNA was isolated from muscle samples disrupted in a Multi-Bead Shocker (M&S Instruments, Osaka, Japan) using an RNeasy Micro Kit (Qiagen). First-strand cDNA was synthesized using a Super Script III First Strand Synthesis System for RT-PCR (Thermo Fisher Scientific). For each PCR assay, 500 ng –1 μg cDNA was used. The primers used in the present study were as follows: *decorin*, forward, 5′-TGCTGCTGCCGTC CATGCTGAT-3′, and reverse, 5′-CATGCCTGGCTGTCCGCA CA-3′; *MyoD*, forward, 5′-GCCGCCTGAGCAAAGTGAATG-3′, and reverse, 5′-CAGCGGTCCAGGTGCGTAGAAG-3′. As an internal control, the primer set used for the housekeeping gene, glyceraldehyde-3-phosphate dehydrogenase (*Gapdh*) was as follows: mouse, forward, 5′- GATGACATCAAGAAGGT GGTGA-3′, and reverse, 5′-TGCTGTAGCCGTATTCATTGTC-3′. Quantitative PCR was performed using SYBR^®^ Premix Ex Taq^TM^ II (Perfect Real Time, Takara Bio Inc., Ohtsu, Japan). SYBR green detection of PCR products was conducted in real time using the MyiQ single-color detection system (Bio-Rad, Hercules, CA).

### Western Blotting Analysis

The proteins were separated by electrophoresis using precast NuPAGE 4–12% Bis-Tris gels (Thermo Fisher Scientific) in NuPAGE^TM^ 3-(*N*-morpholino) propanesulfonic acid buffer (pH 7.7) containing sodium dodecyl sulfate, and then transferred to a polyvinylidene difluoride membrane. The membranes were blocked for 60 min at room temperature in Tris-buffered saline containing 0.1% Tween 20 and 5% skim milk. The blots were probed with the primary antibody: mouse monoclonal antibody to decorin (R&D Systems, clone 115402) or GAPDH (Santa Cruz, Dallas, TX, clone G-4) at 1:1,000 dilution overnight at 4°C or for 60 min at room temperature and then incubated with horseradish peroxidase (HRP)-conjugated goat anti-mouse IgG (Cytiva, Marlborough, MA) at 1:2,000 dilution for 45 min. Immunoreactive proteins were detected and quantified using an enhanced chemiluminescence system, Image Quant LAS 4000 coupled with Image Quant TL software (GE Healthcare, Chicago, IL).

### Proteome Cytokine/Chemokine Array

The relative expression of cytokines and chemokines in muscle lysate was quantified using the Proteome Profiler^TM^ Array (Mouse Cytokine/chemokine Array, Panel A; R&D Systems), as previously described (Nitahara-Kasahara et al., 2014, 2021a). To achieve maximum assay sensitivity, the blots were incubated overnight with the lysate. Enhanced chemiluminescence incubation was performed for 5 min using a Super Signal West Femto Chemiluminescence Kit (Thermo Scientific Pierce), and the samples were imaged and analyzed using Image Quant LAS 4000 coupled with Image Quant TL software (GE Healthcare).

### Quantitative Analysis of Chondroitin Sulfate and Dermatan Sulfate Disaccharides

The disaccharide compositions of the CS and DS moieties of CS/DS hybrid chains in the skeletal muscle of mice were assessed as described previously (Mizumoto and Sugahara, 2012). Briefly, the GAG fraction was crudely purified from the tissue and then digested with a mixture of chondroitinase AC-I and AC-II, or chondroitinase B. Each digest was labeled with a fluorophore, 2-aminobenzamide, and then analyzed using anion-exchange high-performance liquid chromatography (HPLC) on a PA-G silica column (4.6 × 150 mm; YMC Co., Kyoto, Japan). Identification and quantification of the resulting disaccharides were achieved by comparing with the elution positions of the CS- or DS-derived authentic unsaturated disaccharides. The amount of disaccharides in each sample was calculated by comparing the peak areas of standard unsaturated disaccharides.

### Statistical Analyses

Data are presented as the mean ± standard deviation. Differences between two groups were assessed using unpaired two-tailed *t*-tests. Multiple comparisons between three or more groups were performed using a one-way or two-way analysis of variance. Statistical differences were defined as ^∗^*p* < 0.05, ^∗∗^*p* < 0.01, ^∗∗∗^*p* < 0.001, and ^****^p < 0.0001, and were calculated using Excel (Microsoft, Redmond, WA, United States) and GraphPad Prism 8 (GraphPad, La Jolla, CA).

## Results

### Decorin Expression and Localization in the Dermatan Sulfate Deficiency Muscle

**FIGURE 1:**
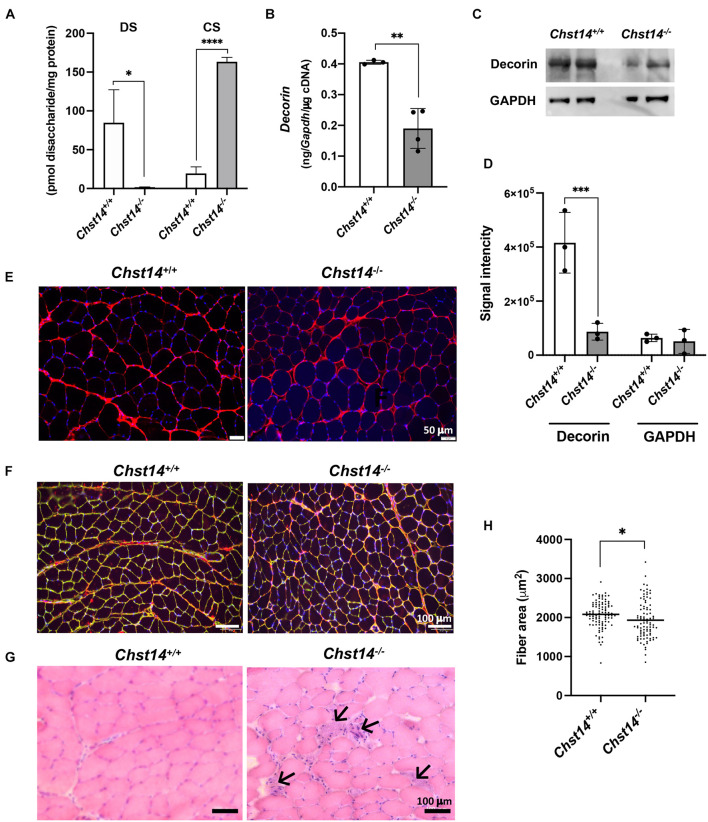
Decorin expression and localization in dermatan sulfate (DS) deficient muscle. **(A)** Total amounts of chondroitin sulfate (CS) and DS disaccharides derived from the tibialis anterior (TA) muscle of *Chst14*^+/+^ and *Chst14^–/–^* mice, analyzed by anion-exchange HPLC after enzymatic digestion. **(B)** *Decorin* mRNA in the TA muscle from *Chst14*^+/+^ (*n* = 3) and *Chst14^–/–^* (*n* = 4) mice was quantified by reverse transcription polymerase chain reaction (RT-PCR). Quantitative data were normalized using the glyceraldehyde 3-phosphate dehydrogenase (*Gapdh*) signal. **(C)** The protein level of decorin in the TA muscle were analyzed by western blotting using anti-decorin antibody and anti-glyceraldehyde 3-phosphate dehydrogenase (GAPDH) antibodies. **(D)** Signal intensity quantified using blotting band images using Image Quant TL software. **(E)** Immunofluorescence staining of TA muscle from *Chst14*^+/+^ and *Chst14^–/–^* mice was performed using anti-decorin antibody (red signals). Nuclear staining was detected using 4,6-diamidino-2-phenylindole (blue signals). Bars, 50 μm. **(F)** Dual staining of the TA muscle from *Chst14*^+/+^ and *Chst14^–/–^* mice for the detection of decorin (red signals) and laminin (green signals) with nuclear staining (blue signals). Bars, 100 μm. **(G)** Hematoxylin and eosin (H&E) staining of the TA muscle from *Chst14*^+/+^ and *Chst14^–/–^* mice. Arrows show the nuclear accumulation and spread of muscle fiber stroma. Bars, 100 μm. **(H)** Muscle fiber areas (μm^2^) measured from the TA muscle of *Chst14*^+/+^ and *Chst14^–/–^* mice using H&E staining. Each fiber area is indicated by a dot, and the average fiber area is described as a bar for each muscle. In total, 1931 fibers are represented in the dot plot. Median values are indicated by red bars. Statistical differences between *Chst14*^+/+^ and *Chst14^–/–^* (*P < 0.05, **P < 0.01. ***P < 0.001, and ****P < 0.0001), *t*-test. All data were analyzed using the TA muscle of 1-year-old sex-matched *Chst14*^+/+^ (*n* = 3) and *Chst14^–/–^* (*n* = 3) mice.

To confirm D4ST1 inactivation for DS biosynthesis in *Chst14^–/–^*mice, we analyzed the amount of CS and DS disaccharides in the skeletal muscle (Figure 1A). Largely suppressed DS disaccharides and an increase in CS disaccharides were observed in *Chst14^–/–^* mice compared to the wild type (*Chst14^+/+^*) mice, suggesting DS deficiency due to D4ST1 inactivation (Supplementary Table 1). To investigate the expression levels and localization of decorin in skeletal muscle, quantitative reverse-transcription PCR, western blotting, and immunohistological analysis were performed using the TA muscle from *Chst14^–/–^* mice. We confirmed that the mRNA expression of decorin was downregulated in the *Chst14^–/–^* mice compared to that in the *Chst14^+/+^* mice (Figure 1B). The expression of glycanated decorin was also downregulated in *Chst14^–/–^* mice compared to that in *Chst14^+/+^* mice, whereas the expression levels of the internal control, GAPDH protein, were not changed based on western blot and quantitative data (Figures 1C,D). In healthy muscle, histochemical images showed that most of the decorin was localized in the perimysium, which is the sheath of connective tissue that covers a bundle of muscle fibers, whereas a small amount was found in the endomysium, a layer of connective tissue that surrounds individual muscle fibers (Figure 1E). In contrast, decorin in the muscle of *Chst14^–/–^* mice was localized in the perimysium around packages of muscle fibers and was augmented around individual muscle fibers in the endomysium (Figure 1E). The decorin in the *Chst14^–/–^* mice was co-localized with laminin in the endomysium, whereas decorin did not co-localization with laminin in the perimysium of muscle from *Chst14^+/+^* mice, as shown in Figure 1F. A cross-section of the TA muscle from *Chst14^–/–^* mice revealed the spreading of the muscle fiber interstitium and cell infiltration by H&E staining (Figure 1G). The histopathological findings observed in *Chst14^–/–^* mice indicated high myofiber size variability due to a higher number of smaller fibers (Figure 1H). Furthermore, central nuclear fibers, which are regenerated fibers that have undergone degeneration, were observed in *Chst14^–/–^* mice (0.96% per total number of fibers), whereas only a small percentage were found in *Chst14^+/+^* mice (0.28%).

### Effect of Chst14 on Myogenesis and Myokine/Chemokine Expression in the Muscle

**FIGURE 2:**
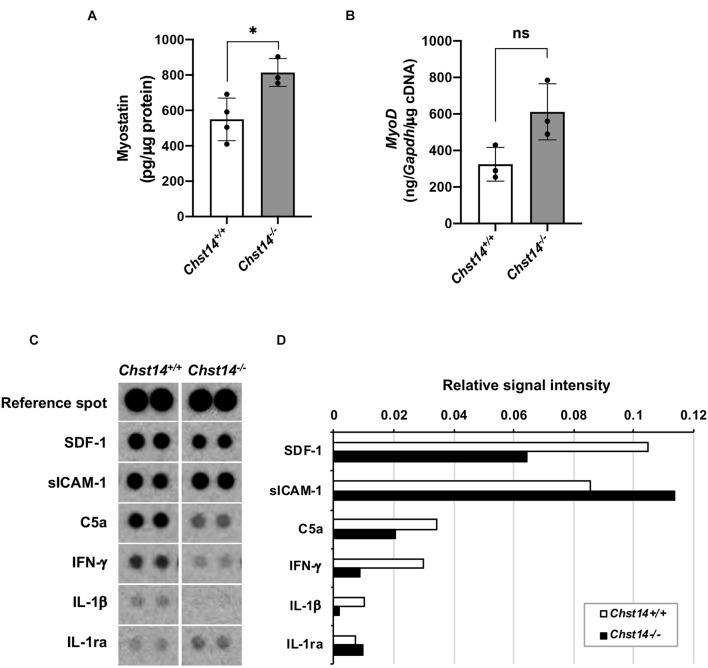
Expression of myogenesis-associated factors and myokines/chemokines in DS-deficient muscle. **(A)** Quantitative measurement of myostatin expression (pg/μg protein) in the muscle lysate using an enzyme-linked immunosorbent assay (ELISA) (*p < 0.05). **(B)** Quantitative measurement of *MyoD* mRNA in the muscle from 1-year-old sex-matched *Chst14^+/+^* (*n* = 8) and *Chst14^–/–^* (*n* = 4) mice by quantitative real-time PCR were normalized with *Gapdh* (ng/*Gapdh*/μg cDNA). Data are presented as mean ± standard deviation, and there was no statistical difference between *Chst14^+/+^* and *Chst14^–/–^* by *t*-test. (ns, not significant). **(C)** Cytokine and chemokine expression in the muscle from 1-year-old sex-matched *Chst14^+/+^* (*n* = 3) and *Chst14^–/–^* (*n* = 3) mice were analyzed using the Proteome profiler^TM^ array. Images of dot signals showed changes in the expression levels of stromal cell-derived factor 1 (SDF-1), soluble intercellular adhesion molecule-1 (sICAM-1), complement component 5a (C5a), interferon-γ (IFN-γ), IL-1β, and IL-1ra, compared to the reference spot signals. **(D)** Relative signal intensity correlated by reference spot signals in the array images were quantified using Image Quant TL software.

mcEDS showed a smaller area of muscle fibers compared to healthy muscle; therefore, we investigated the possibility of muscle formation in the mcEDS and myokine environments. Myostatin, a negative regulator, was upregulated in the muscle of *Chst14^–/–^* mice compared to that in the muscle of *Chst14*^+/+^ mice (Figure 2A). We assessed expression of MyoD, which is a muscle-growth-associating factor that maintains a regulated signal pathway toward muscle growth, but did not find a significant difference in *MyoD* mRNA expression in the muscle between *Chst14*^+/+^ and *Chst14^–/–^* mice (Figure 2B).

To investigate the changes in the myokine and chemokine expression in the DS-deficient muscle, we performed a cytokine/chemokine array using the muscle lysate (Figures 2C,D). Quantitative results demonstrated that soluble intercellular adhesion molecule-1 (sICAM-1) showed strong signals in the muscles of both *Chst14^+/+^* and *Chst14^–/–^* mice. Stromal cell-derived factor 1 (SDF1), complement component C5a, and pro-inflammatory cytokines, interferon-γ (IFN-γ) as well as IL-1β, was found to be reduced in *Chst14^–/–^* mice. In contrast, IL-1ra, an antagonist of IL-1, was slightly increased compared to that in the *Chst14^+/+^* mice (Figures 2C,D), suggesting an altered cytokine/chemokine balance in DS-deficient muscles.

### Enhanced Fibrosis in the Dermatan Sulfate-Deficient Muscle

**FIGURE 3:**
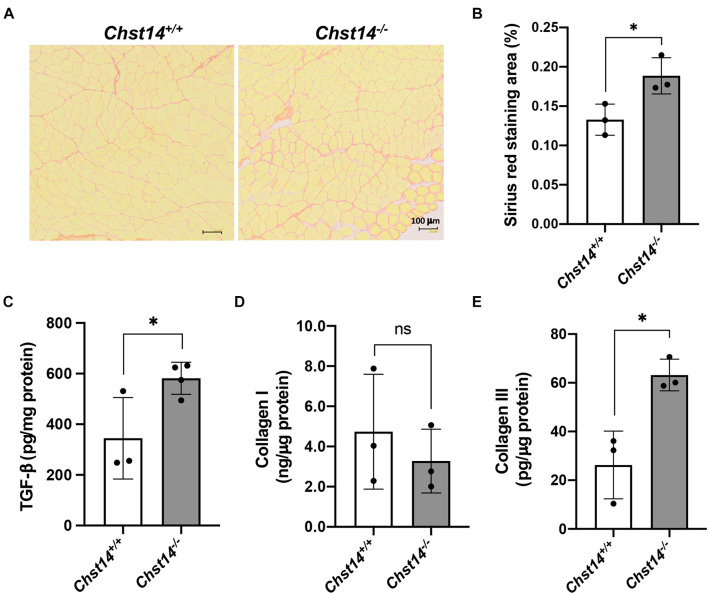
Fibrosis in DS-deficient muscles. **(A)** Sirius red staining of the tibialis anterior (TA) muscle of 1-year-old sex-matched *Chst14^+/+^* and *Chst14^–/–^* mice. Bars, 100 μm. **(B)** Quantification of sirius red staining area of the cross-section (% of total area) from 1-year-old sex-matched *Chst14^+/+^* and *Chst14^–/–^* mice (*n* = 3, each). **(C–E)** Quantitative measurement of TGF-β **(C)**, collagen type I **(D)**, and collagen type III **(E)** in the muscle lysate from 1-year-old sex-matched *Chst14^+/+^* and *Chst14^–/–^* (*n* = 3, each) mice using ELISA. All data are presented as mean ± standard deviation, and statistical differences between *Chst14^+/+^* and *Chst14^–/–^* (*P < 0.05), *t*-test. ns, not significant.

Decorin can interact with fibrillar collagens and is assumed to play a role in fibril formation and the maintenance of the fibrillar network, organizing the ECM (Toole and Lowther, 1968; Weber et al., 1996). To examine disease-associated fibrosis in DS-deficient muscle, we performed sirius red staining (Figures 3A,B). *Chst14^–/–^* mice showed a higher fibrotic area in the muscle compared to that in *Chst14^+/+^* mice. To further characterize fibrosis in mcEDS, the expression of TGF-β1 and collagen type I and III, which are classically upregulated in fibrotic processes, was measured by ELISA. We confirmed the upregulation of TGF-β1 and collagen type III, but not of collagen type I (Figures 3C–E). These results suggest that fibrosis is enhanced in DS-deficient muscle.

## Discussion

**FIGURE 4:**
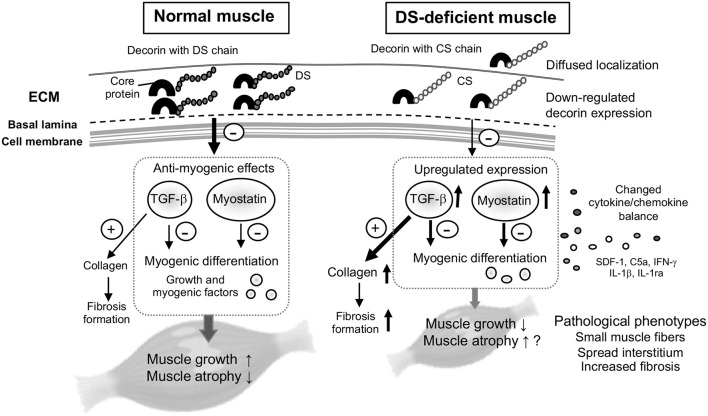
Schematic for the proposed hypothesis of this study. Decorin is one of the most abundant proteoglycans in the skeletal muscle, mainly associated with ECM surrounding bundles of myofibers. In normal muscle, decorin acts as a myokine through the pathway: inhibition of transforming growth factor (TGF)-β, and down-regulation of myostatin expression, by competitively binding to TGF-β or myostatin. The inhibitory effects on TGF-β or myostatin (arrow with minus mark) might be reduced in DS-deficient muscle, because down-regulation of decorin core protein as well as its modification by CS instead of DS lead to diffused localization and induced up-regulated expression of myostatin and TGF-β, with the resulting collagen production leading to fibrosis. Decorin with CS also leaded to changes in the cytokine/chemokine environment, and might result in delayed muscle growth and pathological phenotypes, but had no effect on expression levels of myogenic factors.

In the present study, we investigated the effects of DS deficiency on myogenesis and the potential cause of myopathy. We demonstrated that the pathological decorin localization and functional abnormalities of decorin with the CS side chain were caused by DS deficiency in mcEDS, which causes disturbances in myogenesis of skeletal muscle, suggesting disease-specific phenotypes in myogenesis. Figure 4 summarizes the hypotheses proposed in this study.

We focused on the pathology analysis to eliminate the effects of decreased motor function in mcEDS mice in the present study (Nitahara-Kasahara et al., 2021b), even though decorin has been reported to act as a myokine after exercise (Kanzleiter et al., 2014). In *Chst14^–/–^* mice, a decrease of DS accompanied with an increase of CS was observed in TA muscle (Figure 1). Furthermore, total CS/DS was also increased in the *Chst14*-deficient mice, indicating that the chain length as well as number of CS may be increased in the mice. Most of the decorin with DS chains was localized in the perimysium of normal muscle; however, decorin with the CS chain showed co-localization with laminin and was diffused in the perimysium and endomysium (Figure 1). In the muscle of *Chst14^–/–^* mice, decreased expression and altered localization of decorin core protein may affect the conventional functions of decorin. We previously reported the structural and conformational alteration of GAG chains of decorin-proteoglycan in the skin of patients with mcEDS-*CHST14* (Hirose et al., 2019). By electron microscopy staining, rod-shaped linear GAG chains were found to be attached at one end to collagen fibrils and protruded outside the fibrils in the skin of mcEDS-*CHST14*, in contrast to those in wild type mice where they surround and wrap the collagen fibrils. The structure of the GAG chain from *Chst14^–/–^* mice also exhibited similar abnormalities of collagen networks in the skin (Hirose et al., 2021). Similar to these findings observed in skin tissue, structural and conformational abnormalities in the GAG chain on decorin may affect the formation of collagen fibrils in the muscle tissue. Decorin-null mice exhibit dermal collagen fibrils with a large variety of sizes and shapes (Danielson et al., 1997). These findings suggest that the decorin-proteoglycan is important for collagen fibril formation, and regulates the space between collagen fibrils as well as bundles, as reported previously (Scott et al., 1995). DS and CS/DS hybrid chains are conformationally more flexible than CS chains (Casu et al., 1988; Hirose et al., 2021). Thus, collagen bundles bound by CS chains, instead of DS chains, on decorin in mcEDS-*CHST14* patients as well as *Chst14^–/–^* mice may be more fragile than those in healthy and wild-type controls, respectively.

The diffused decorin localization in the spread of the endomysium and perimysium of DS-deficient muscle was similar to that of the dystrophic muscle (Caceres et al., 2000). In dystrophic skeletal muscle, the biosynthesis and accumulation of decorin around individual muscle fibers are enhanced in the endomysium and exomysium (Caceres et al., 2000). The histopathological findings observed in the *Chst14^–/–^* mice indicated that several central nuclear fibers, nuclear infiltration, and fibrosis were not as high as in severe myopathy and dystrophy (Figure 1; Coulton et al., 1988). Although dystrophic muscles showed a larger number of smaller fibers, occurrence of hypertrophic fibers, and high levels of creatine kinase, a marker of muscle damage, this was not confirmed in *Chst14^–/–^* mice, suggesting milder phenotypes of myopathy in mcEDS.

Altered localization of decorin indicates structural modification of the ECM in the spreading interstitium of the DS-deficient muscles. Expression of decorin mRNA was confirmed in the connective tissue cells, that is, in the mesenchymal and satellite cells, suggesting that decorin plays an important role in organizing the fibrillar network of the ECM (Caceres et al., 2000; Fadic et al., 2006). In the muscle of *Chst14^–/–^*mice, upregulated myostatin appears to induce muscle growth delay and muscle dystrophy. MyoD and follistatin have also been reported to increase in response to decorin overexpression (Kanzleiter et al., 2014). *Chst14^–/–^* mice had decorin with a CS side chain and showed lower activity of myogenesis and muscle formation in the DS-deficient muscle, supported by a larger number of smaller fibers, while the significant difference of *MyoD* expression was not detected between *Chst14^–/–^* and *Chst14^+/+^* mice.

Besides function as a matrix component, biglycan-proteoglycan can be either proteolytically released from the ECM upon tissue stress and injury or synthesized by activated macrophages (Schaefer et al., 2005). Biglycan protein core together with GAG side chain(s), triggers a proinflammatory response by acting as a signaling molecule and an endogenous ligand of Toll-like receptors (TLR)-2 and -4 on the surface of macrophages. It then induces the synthesis and secretion of pro-inflammatory cytokines and chemokines, such as IL-1β, tumor necrosis factor (TNF)-α, chemokine (C-C motif) ligand (CCL) 2 and 5, and chemokine (C-X-C motif) ligand (CXCL)1, -2, and -13 (Schaefer et al., 2017). These processes initiate modulation of the immune environment. Pathogenic muscle-derived cytokines are thought to be produced by infiltrating inflammatory cells. Cytokine and chemokine arrays demonstrated that the expression pattern of SDF-1, IFN-γ, C5a, IL-1β, and IL-1ra was altered in *Chst14^–/–^* mice (Figure 2). Thus, the reduction of DS in *Chst14^–/–^* mice might affect the expression and/or stability of cytokines as well as chemokines in the ECM or on the cell surface. SDF-1 and its receptor CXCR4 and CXCR7, stimulate the production of paracrine mediators, including some of growth factors such as vascular endothelial growth factor, fibroblast growth factor, and hepatocyte growth factor (Liu et al., 2012) associated muscle growth. Thus, the reduction of SDF-1 may indicate lower activity of growth factors in *Chst14*^–/–^ mice. C5a, which is known to play recruitment of inflammatory cells, and lead to pro-inflammatory cytokines. Thus, low expression of C5a may suppress pro-inflammatory activation in *Chst14*^–/–^ mice. The *IL-1ra* gene has been associated with various human diseases, primarily epithelial and endothelial cells. This may indirectly lead to an imbalance in the IL-1 system with enhanced production of IL-1β and reduced production of IL-1ra (Arend, 2002). In DS-deficient muscle, disease-specific changes in cytokine balance could affect myogenesis and induce disease progression. Functional changes in CS-containing decorin may induce a modified environment for some myokines.

The ECM is essential for normal myogenesis, which includes interactions between myoblasts and their environment (Osses and Brandan, 2002). Decorin plays an important role in organizing the fibrillar network of the ECM (Caceres et al., 2000; Fadic et al., 2006). Various proteoglycans in the ECM have been reported to play a role in the differentiation process by regulating growth factor activity (Villena and Brandan, 2004). DS is an enhancer of growth factor-dependent proliferation of satellite cells and migration during skeletal muscle formation (Villena and Brandan, 2004). Therefore, DS depletion in the skeletal muscle of *Chst14*^–/–^ mice and mcEDS may induce disease-specific myogenesis, including delayed muscle growth and reduced structural stability.

CS/DS-proteogrycans regulate cell signaling on the cell surface through binding with various growth factors (Mizumoto et al., 2015). Both the amount and distribution of iduronic acid (IdoUA) are subjected to physiological regulation; for example, TGF-β considerably affects IdoUA in decorin (Tiedemann et al., 2005). The conformational flexibility of IdoUA-containing CS/DS hybrid as well as DS chains is thought to facilitate the binding activity to proteins (Casu et al., 1988). For instance, the interaction of hepatocyte growth factor with CS/DS requires IdoUA residue flanked by 4-*O*-sulfated *N*-acetylgalactosamine (Deakin et al., 2009). The IdoUA-containing domains of CS/DS have also been shown to interact with the fibroblast growth factor family, thereby regulating cell migration (Trowbridge et al., 2002). Considering these facts, the IdoA residue seems to effects of the replacement of DS by CS migration and proliferation of muscle cells in *Chst14*^–/–^ mice.

Decorin shows high affinity for TGF-β by binding to decorin core protein (Hildebrand et al., 1994), allowing decorin to function as a reservoir for TGF-β in the ECM. We demonstrated enhanced fibrosis in *Chst14*^–/–^ mice supported by histopathological staining and upregulated expression of collagen and TGF-β (Figure 3). Decorin with the CS chain in *Chst14*^–/^*^–^* mice led to enhanced fibrosis and resulted in connective tissue fragility, as observed in dystrophic muscle. In patients with dystrophy, selective accumulation of CS, increase in 4-*O*-sulfation of CS accompanied by upregulation of *CHST11*, which encodes chondroitin 4-*O*-sulfotransferase-1, and reduction in expression of CS-degrading enzyme, hyaluronidase-4, in the muscles (Negroni et al., 2014). In this study, we demonstrated that DS chain of decorin-proteoglycan was replaced with CS, and that its protein expression was reduced in *Chst14^–/–^* mice (Figure 1). The functional alteration by decorin-proteoglycan in *Chst14*^–/–^ mice might lead the myopathy phenotype in the muscle. Further investigation may be required for more understanding of the mechanisms of myopathy phenotype caused by decorin-proteoglycan with CS side chain.

Abnormal collagen bundle formation was associated with decorin GAG abnormalities. Decorin interacts with collagen I as well as with collagens II, III, IV, V, VI, XII, and XIV (Gubbiotti et al., 2016). The fibril-forming of types I and III are by far the most abundant by proteomic studies, suggesting that they jointly account for approximately 75% of total muscle collagen (McKee et al., 2019). The strong parallel fibers of collagen I, which are present in the endo-, peri-, and epimysium, are assumed to confer tensile strength and rigidity to the muscle, whereas collagen III forms a loose meshwork of fibers that bestows elasticity to the endo- and perimysium (Kovanen, 2002). Our data showed downregulation of collagen III in the muscle of *Chst14^–/^*^–^ mice. Therefore, the loose meshwork of fibers might be associated with the myopathy phenotype in *Chst14^–/^*^–^ mice, and mcEDS may be caused by connective tissue fragility in the skeletal muscle, associated with ECM functional changes including ectopic localization of decorin. These findings will facilitate future research on the disease-specific mechanisms of decorin with DS or CS chains in muscle maintenance and potential therapeutic approaches for myopathy.

## Data Availability Statement

The datasets presented in this study can be found in online repositories. The names of the repository/repositories and accession number(s) can be found below: Gene ID: 16176, https://www.ncbi.nlm.nih.gov/gene/16176; Gene ID: 16181, https://ww w.ncbi.nlm.nih.gov/gene/16181; Gene ID: 14433, https://www. ncbi.nlm.nih.gov/gene/14433; Gene ID: 17927, https://www.ncbi. nlm.nih.gov/gene/17927; and Gene ID: 13179, https://www.ncbi. nlm.nih.gov/gene/13179.

## Ethics Statement

The animal study was reviewed and approved by the Experimental Animal Care and Use Committee at the National Center of Neurology and Psychiatry (NCNP) and the Nippon Medical School.

## Author Contributions

YN-K and TK conceived and planned the experiments. YN-K, SM, YI, GP-H, and AN-T performed the experiments, derived the models, contributed to sample preparation, assisted with experiments involving animal models, and analyzed the data. SM, TI, SY, YN, and ST provided advice and suggestions for the discussion. YN-K wrote the manuscript in consultation. TO and TK supervised the project. All authors contributed to the article and approved the submitted version.

## Conflict of Interest

The authors declare that the research was conducted in the absence of any commercial or financial relationships that could be construed as a potential conflict of interest. The reviewer FM declared a past co-authorship with several of the authors SM, SY, TK to the handling editor.

## Publisher’s Note

All claims expressed in this article are solely those of the authors and do not necessarily represent those of their affiliated organizations, or those of the publisher, the editors and the reviewers. Any product that may be evaluated in this article, or claim that may be made by its manufacturer, is not guaranteed or endorsed by the publisher.
