# Supplementary material for: Myopathy Associated With Dermatan Sulfate-Deficient Decorin and Myostatin in Musculocontractural Ehlers-Danlos Syndrome: A Mouse Model Investigation
Source: Front Cell Dev Biol. 2021 Oct 11;9:695021. doi: 10.3389/fcell.2021.695021 (PMC8542786; doi:10.3389/fcell.2021.695021)
Supplement: Supplementary file 1 [file Table_1.docx]

**Supplementary Table S1. Disaccharide composition of the CS and DS chains from tibialis anterior muscles.**

The GAG-peptides fractions prepared from tibialis anterior muscles of *Chst14^+/+^*, *Chst14^+/–^*, and *Chst14^–/–^* mice with a nucleotide deletion by CRISPR/Cas9 were individually digested with a mixture of chondroitinases AC-I (Yamagata et al., 1968) and AC-II (Hiyama and Okada, 1975) or chondroitinase B (Michelacci and Dietrich, 1974) for analysis of CS or DS moiety, respectively, and the each digest was labeled with 2-aminobenzamide (2AB) and analyzed by anion-exchange HPLC (Kinosita and Sugahara, 1999; Nitahara-Kasahara et al., 2021). The amount of resultant disaccharides in each sample was calculated based on the peak area in each chromatogram (Nitahara-Kasahara et al., 2021).

| **CS moiety** | *Chst14^+/+^* | *Chst14^+/–^* | *Chst14^–/–^* |
| --- | --- | --- | --- |
|  | pmol disaccharide/mg total protein | | |
| ∆HexUA-GalNAc | N.D. | N.D. | N.D. |
| ∆HexUA-GalNAc(6S) | 7.1 ± 1.3 | 4.7 ± 0.7 | 9.6 ± 0.3* |
| ∆HexUA-GalNAc(4S) | 12.3 ± 5.7 | 20.6 ± 0.7 | 153.6 ± 3.1** |
| **Total CS disaccharide** | **19.4 ± 4.9** | **25.4 ± 1.4** | **163.2 ± 3.3**** |

| **DS moiety** | *Chst14^+/+^* | *Chst14^+/–^* | *Chst14^–/–^* |
| --- | --- | --- | --- |
|  | pmol disaccharide/mg total protein (mol%) | | |
| ∆HexUA-GalNAc | N.D. | N.D. | N.D. |
| ∆HexUA-GalNAc(6S) | N.D. | N.D. | N.D. |
| ∆HexUA-GalNAc(4S) | 68.2 ± 16.0 | 116.3 ± 25.1 | 1.7 ± 0.2*** |
| ∆HexUA(2S)-GalNAc(4S) | 25.0 ± 4.0 | 34.50 ± 8.1 | N.D. |
| T**otal DS disaccharide** | **84.8 ± 24.5** | **150.7 ± 33.1** | **1.7 ± 0.2†** |

Three mice were utilized for each analysis.

N.D., not detected (<1 pmol disaccharide/mg total protein)

*p<0.05 (Student’s *t*-test, *Chst14^+/+^* or *Chst14^+/–^* vs. *Chst14^–/–^*); **p<0.0001; ***p<0.02; †p<0.05 (*Chst14^+/+^* or *Chst14^+/–^* vs. *Chst14^–/–^*).

Abbreviations: ∆HexUA, 4,5-unsaturated hexuronic acid; GalNAc, *N*-acetyl-D-galactosamine;

2S, 4S, and 6S, 2-*O*-, 4-*O*-, and 6-*O*-sulfate, respectively.

**References for Supplementary Materials**

Hiyama, K., and Okada, S. (1975). Crystallization and some properties of chondroitinase from *Arthrobacter aurescens*. *J. Biol. Chem.* 250, 1824-1828.

Kinoshita, A., and Sugahara, K. (1999). Microanalysis of glycosaminoglycan-derived oligosaccharides labeled with a fluorophore 2-aminobenzamide by high-performance liquid chromatography: application to disaccharide composition analysis and exosequencing of oligosaccharides. *Anal. Biochem*. 269, 367-378.

Michelacci, Y. M., and Dietrich, C. P. (1974). Isolation and partial characterization of an induced chondroitinase B from *Flavobacterium heparinum*. *Biochem. Biophys. Res. Commun*. 56, 973-980.

Nitahara-Kasahara, Y., Mizumoto, S., Inoue, Y. U., Saka, S., Posadas-Herrera, G., Nakamura-Takahashi, A., Takahashi, Y., Hashimoto, A., Konishi, K., Miyata, S., Masuda, C., Matsumoto, E., Maruoka, Y., Yoshizawa, T., Tanase, T., Inoue, T., Yamada, S., Nomura, Y., Takeda, S., Watanabe, A., Kosho, T., and Okada, T. (2021). Muscle pathophysiology in mouse models of musculocontractural Ehlers-Danlos syndrome due to *CHST14* mutations (mcEDS-*CHST14*), generated through CRISPR/Cas9-mediated genomic editing. *Disease Models and Mechanisms* Submitted.

Yamagata, T., Saito, H., Habuchi, O., and Suzuki, S. (1968). Purification and properties of bacterial chondroitinases and chondrosulfatases. *J. Biol. Chem*. 243, 1523-1535.
